# Neflamapimod induces vasodilation in resistance mesenteric arteries by inhibiting p38 MAPKα and downstream Hsp27 phosphorylation

**DOI:** 10.1038/s41598-022-08877-8

**Published:** 2022-03-22

**Authors:** Ajay K. Pandey, Farzana Zerin, Sreelakshmi N. Menon, Tanzia I. Tithi, Khue P. Nguyen, Tran Vo, Morgan L. Daniel, Sherif Hafez, Md. Ashraful Alam, Raquibul Hasan

**Affiliations:** 1 Department of Pharmaceutical Sciences, College of Pharmacy, Mercer University, 3001 Mercer University Drive, Atlanta, GA 30341 USA; 2 Department of Pharmaceutical Sciences, North South University, Dhaka, 1229 Bangladesh

**Keywords:** Drug discovery, Physiology, Cardiology

## Abstract

Neflamapimod, a selective inhibitor of p38 mitogen activated protein kinase alpha **(**MAPKα), is under clinical investigation for its efficacy in Alzheimer’s disease (AD) and dementia with Lewy Bodies (DLB). Here, we investigated if neflamapimod-mediated acute inhibition of p38 MAPKα could induce vasodilation in resistance-size rat mesenteric arteries. Our pressure myography data demonstrated that neflamapimod produced a dose-dependent vasodilation in mesenteric arteries. Our Western blotting data revealed that acute neflamapimod treatment significantly reduced the phosphorylation of p38 MAPKα and its downstream target heat-shock protein 27 (Hsp27) involved in cytoskeletal reorganization and smooth muscle contraction. Likewise, non-selective inhibition of p38 MAPK by SB203580 attenuated p38 MAPKα and Hsp27 phosphorylation, and induced vasodilation. Endothelium denudation or pharmacological inhibition of endothelium-derived vasodilators such as nitric oxide (NO) and prostacyclin (PGI_2_) had no effect on such vasodilation. Neflamapimod-evoked vasorelaxation remained unaltered by the inhibition of smooth muscle cell K^+^ channels. Altogether, our data for the first time demonstrates that in resistance mesenteric arteries, neflamapimod inhibits p38 MAPKα and phosphorylation of its downstream actin-associated protein Hsp27, leading to vasodilation. This novel finding may be clinically significant and is likely to improve systemic blood pressure and cognitive deficits in AD and DLB patients for which neflamapimod is being investigated.

## Introduction

p38, a member of the mitogen-activated protein kinase (MAPK) family, is traditionally known to be a stress sensor that is activated by physical and chemical stressors, inflammatory mediators, vasoactive compounds, and growth factors^1–4^. Among the four isoforms of p38 MAPK, namely α, β, γ and δ^5,6^, p38 MAPKα is present in most cell types and is best characterized for its role in chronic inflammatory processes linked to degenerative diseases of the nervous system, musculoskeletal system, and cardiovascular system^7^. In microglia, p38 MAPKα activation is associated with the release of pro-inflammatory cytokines. Stimulation of p38 MAPKα signaling in neurons regulates tau localization and neuronal plasticity^8–10^. In addition, p38 MAPKα regulates the function of a Ras-related protein Rab5, which is a key regulator of early endosomal trafficking involved in a wide variety of cell signaling and cellular processes. Due to the involvement of p38 MAPKα in different neuronal pathologies, p38 MAPKα inhibitors have recently attracted considerable attention as potential therapeutics for inflammatory and neurodegenerative diseases, such as Alzheimer’ Disease (AD), Dementia with Lewy Bodies (DLB) and Huntington’ Disease (HD)^11,12^. Neflamapimod, a selective inhibitor of p38 MAPKα, is under clinical investigation for its efficacy in improving cognitive deficits in AD and DLB. Preclinical and recent phase II clinical trial data show that neflamapimod treatment is effective in reversing cognitive decline in DLB^13^, however, its efficacy in reversing AD-related cognitive deficiencies remains somewhat unclear. Although the exact mechanism is not fully known, it is established that hypertension and vascular dysfunction drive neurodegeneration and cognitive dysfunction^14^. Hypertension and altered vascular tone are implicated in vascular cognitive impairment that encompasses all sorts of cognitive deficits arising from vascular dysfunction^14–17^. Since vascular tone is a key regulator of peripheral resistance and systemic blood pressure, drugs that relax small resistance arteries are expected to lower systemic blood pressure and enhance cognitive function.

Although p38 MAPKα has been extensively studied in the context of neuroinflammation and neurodegeneration, little is known about its role in vascular contractility. According to a previous study^18^, high-glucose or diabetes activates p38 MAPK in rat aortic smooth muscle cells. As p38 MAPK activation often reflects gene expression changes leading to cell growth or apoptosis^19–21^, glucose- or diabetes-associated activation of p38 MAPK was suggested to regulate vascular inflammation, thrombosis and other pathological changes^4,22^. Subsequent studies reported that p38 MAPK activation also regulates the contractility of resistance arteries. Vasoconstrictors such as angiotensin II and norepinephrine were reported to cause p38 MAPK activation leading to smooth muscle contraction^7,23,24^. This raises the possibility that acute inhibition of p38 MAPK could stimulate vasodilation, which could reduce vascular resistance and systemic blood pressure. Non-selective p38 MAPK inhibition by SB203580 relaxed high K^+^-contracted renal arteries and enhanced acetylcholine-induced vasodilation^4^. It has been suggested that p38 MAPK activation may influence the function of the cardiovascular system by regulating systemic and renal hemodynamics. To our knowledge, no previous studies have investigated what effect selective p38 MAPKα inhibition by neflamapimod may have on the contractility of resistance mesenteric arteries. Here, we examined if neflamapimod could relax resistance-size rat mesenteric arteries and its mechanism of action. Our data demonstrates that acute neflamapimod treatment stimulates dose-dependent vasodilation in mesenteric arteries, in a manner consistent with p38 MAPKα inhibition and reduced Hsp27 phosphorylation, and independent of endothelial signaling and smooth muscle cell K^+^ channels. Such a novel vasodilatory action of neflamapimod may be clinically translatable and is likely to lower systemic blood pressure and improve cognitive deficits in AD and DLB patients.

## Results

### Neflamapimod stimulates vasodilation in resistance mesenteric arteries

**Figure 1:**
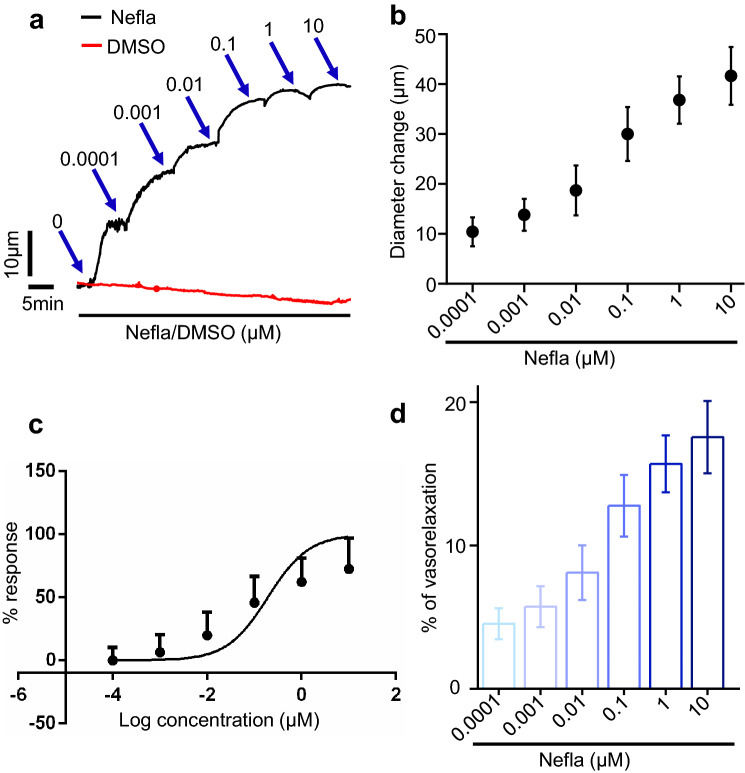
Neflamapimod stimulates vasodilation in resistance-size mesenteric arteries. (**a**) Original traces illustrating a concentration-dependent vasodilation in mesenteric arteries induced by acute neflamapimod (nefla) application (0.0001–10 µM) but not by the vehicle DMSO. Arrows indicate the points of diameter reading for analysis. (**b**) Mean data for diameter changes (over baseline) after the application of increasing concentrations of neflamapimod. (**c**) A sigmoidal curve illustrating concentration–response relationships between the log concentrations of neflamapimod-induced arterial vasodilation. (**d**) Vasorelaxation expressed as % of baseline active diameter at 80 mmHg. n = 5.

To examine if acute neflamapimod treatment could regulate arterial contractility, we performed pressurized arterial myography on resistance 3rd to 5th order mesenteric arteries from male Sprague Dawley rats. Cannulated arterial segments were maintained in a temperature-controlled perfusion chamber (Living Systems), continuously perfused with 37 °C PSS, gassed with a mixture of 21% O_2_, 5% CO_2_, and 74% N_2_. Intravascular pressure was gradually increased to 80 mmHg to stimulate the development of myogenic tone. At 80 mmHg, mesenteric arteries developed ~ 24% myogenic tone, after which a cumulative concentration response (0.0001–10 µM) to neflamapimod was performed and diameter changes recorded. Our myography data shows that, within 2–3 min of application, neflamapimod produces a fully reversible and concentration-dependent vasodilation (Fig. 1a–d). The calculated EC_50_ of neflamapimod for inducing vasodilation was found to be ~ 0.2 µM (Fig. 1c). Of note, the C_max_ reported in a preclinical study with rats is ~ 2 µM^11^. Neflamapimod at 10 µM concentration potently relaxed mesenteric arteries by ~ 41 µm, or by ~ 17% of arterial diameter from a baseline diameter at 80 mmHg (Fig. 1d). Considering the reported C_max_ in a previous study, and for a better diameter read out in the presence of vasomotion, we used 10 µM concentration for subsequent mechanistic studies. Altogether, our data for the first time demonstrate that acute neflamapimod application induces vasodilation in resistance-size mesenteric arteries.

### Neflamapimod inhibits p38 MAPKα phosphorylation in mesenteric arteries

**Figure 2:**
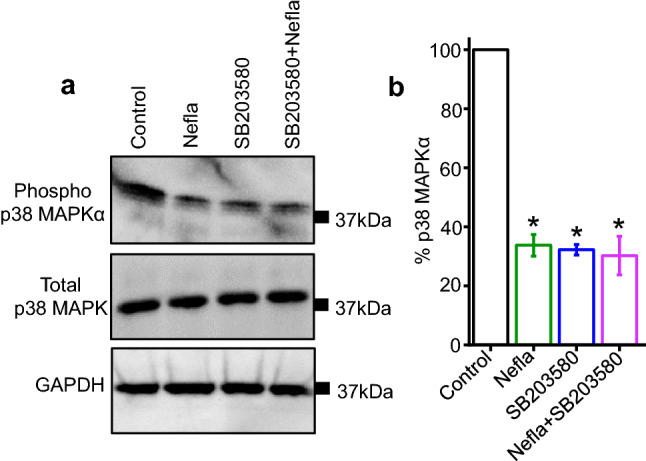
Neflamapimod (nefla) treatment reduces phosphorylation of p38 MAPKα in mesenteric arteries. (**a**) Western blot images showing the modulation of p-38 MAPKα phosphorylation but not total p-38 MAPK by nefla (10 µM), or SB203580 (10 µM) alone or together. Full-length Western blot images are presented in Suppl. Fig. 1. Mesenteric arteries were treated as indicated for 30 min and processed for Western blotting. (**b**) Bar graph comparing p-38 MAPKα phosphorylation normalized to GAPDH. n = 5.

As neflamapimod is a selective inhibitor of p38 MAPKα, we asked if neflamapimod could inhibit p38 MAPKα phosphorylation and its kinase activity, in mesenteric arteries to stimulate vasodilation. To do so, we incubated mesenteric arteries for 30 min with either neflamapimod or SB203580, a non-selective inhibitor of p38 MAPK. Our Western blotting data reveals that mesenteric arteries have a basal p38 MAPKα phosphorylation, as detected by phospho-p38 MAPKα antibody (Fig. 2a, vehicle control, lane 1). Importantly, neflamapimod and SB203580 reduced p38 MAPKα phosphorylation to 33.74 ± 0.3.7% and 32.25 ± 0.1.8% of vehicle-treated control, respectively (Fig. 2a,b). The level of total p38 MAPK was not altered by neflamapimod or SB203580 treatment. These data suggest that both drugs are equally effective in reducing basal p38 MAPKα phosphorylation without altering the expression of total p38 MAPK. Overall, this data demonstrates that mesenteric arteries have baseline p38 MAPKα phosphorylation, and treatment with neflamapimod potently suppresses p38 MAPKα phosphorylation and, therefore, its activity in mesenteric arteries.

### Neflamapimod reduces phosphorylation of actin-associated protein Hsp27

**Figure 3:**
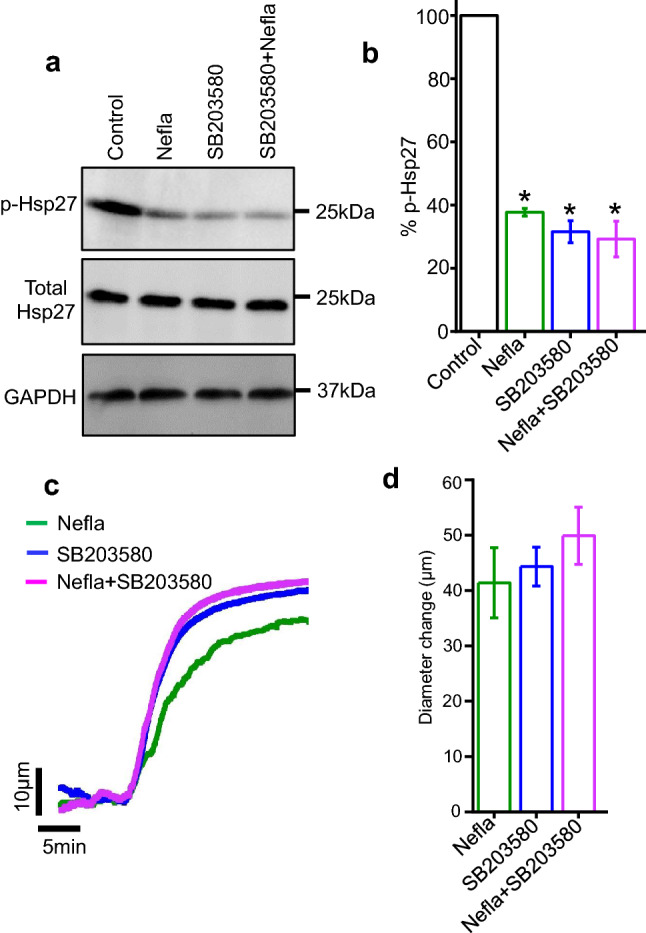
Neflamapimod treatment reduces phosphorylation of Hsp27 in mesenteric arteries. (**a**) Western blot images illustrating decreases in Hsp27 phosphorylation as a function of upstream p38 MAPKα inhibition by nefla (10 µM), or SB203580 (10 µM) alone or in combination. Full-length Western blot images are presented in Suppl. Fig. 2. (**b**) Mean data comparing % of Hsp27 phosphorylation normalized to GAPDH. n = 5. (**c**) Original traces illustrating mesenteric artery vasodilation by selective and non-selective MAPKα inhibitors nefla and SB203580, respectively (**d**) Bar graph comparing mesenteric artery vasodilation. n = 5.

A critical downstream target of p38 MAPK is Hsp27 which, when phosphorylated, promotes actin filament formation and smooth muscle contraction. Earlier studies reported that reduction of Hsp27 phosphorylation by non-selective p38 MAPK inhibition with SB203580 stimulates vasodilation in mesenteric arteries^7,23,24^. This led us to test the hypothesis that neflamapimod-mediated selective inhibition of p38 MAPKα may also decrease Hsp27 phosphorylation to elicit vasodilation. To test this, we processed mesenteric arteries that had been treated with either neflamapimod or SB203580 for Western blotting. Our data revealed that neflamapimod-treated arteries had mean 37.75 ± 1.19% phosphorylation of Hsp27, or 62.25% less compared to that in control arteries (Fig. 3a,b). Likewise, non-selective p38 MAPK inhibitor SB203580 also reduced arterial Hsp27 phosphorylation to 31.57 ± 3.47% or by 69%. Combined application of neflamapimod and SB203580 reduced Hsp 27 phosphorylation to 29.5% ± 5.66%, or by 71% (Fig. 3a,b), which is similar to the inhibition produced by either drugs alone. These data suggest that neflamapimod-induced selective inhibition of p38 MAPKα reduces the phosphorylation of its downstream target Hsp27, an actin-associated protein that takes part in cytoskeletal reorganization and smooth muscle contraction.

We next asked if the inhibition of basal Hsp27 phosphorylation by selective p38 MAPKα inhibitor neflamapimod and non-selective p38 MAPK inhibitor SB203580 correlate with their vasodilatory actions in mesenteric arteries. Our pressure myography data showed that, like neflamapimod, SB203580 application relaxed mesenteric arteries by ~ 44 µm, which is slightly higher than that produced by neflamapimod (Fig. 3c,d). When neflamapimod and SB203580 were applied together, they produced a mean arterial dilation of ~ 50 µm. Importantly, these data correlate with the magnitude of reduction of Hsp27 phosphorylation in mesenteric arteries. Overall, our data demonstrate that neflamapimod selectively inhibits p38 MAPKα and downstream Hsp27 phosphorylation to induce vasodilation in resistance mesenteric arteries.

### Neflamapimod-induced vasodilation does not depend on endothelial signaling

**Figure 4:**
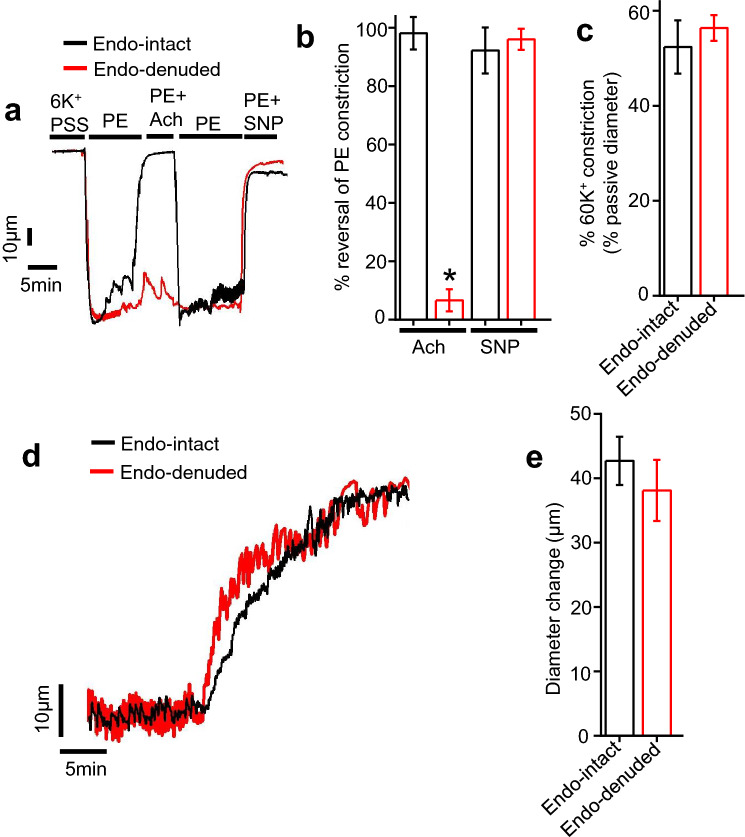
Role of endothelium (endo) in neflamapimod-induced vasodilation. (**a**) Original traces showing diameter changes by 1 µM phenylephrine (PE) alone or in the presence of 1 µM acetylcholine (ACh) or 10 µM sodium nitroprusside (SNP) in endothelium-intact and -denuded mesenteric arteries. (**b**) Mean data showing % reversal of PE-induced vasoconstriction by ACh and SNP, where there is a selective loss of ACh-induced vasodilation in endothelium-denuded vessels. (**c**) Mean data comparing 60 K-induced vasoconstriction in endothelium-intact and -denuded arteries. (**d**) Original traces showing that both endothelium-intact and -denuded arteries have similar magnitude of vasodilation to acute neflamapimod (10 µM) exposure. (**e**) Mean data for neflamapimod-evoked vasodilation in endothelium-intact and -denuded mesenteric arteries. n = 5, *P < 0.05 versus endo-intact.

Endothelium is an important regulator of arterial contractility. Endothelial cells induce vasodilation primarily through the production of vasodilators and their paracrine action on underlying smooth muscle cells. Therefore, we next assessed the role of endothelial signaling in neflamapimod-elicited vasodilation. To determine the role of endothelium, neflamapimod-induced vasodilatory responses in endothelium-intact and -denuded mesenteric arteries were compared. To this end, we performed endothelium denudation by slowing passing air bubbles through the vessel lumen and confirming the absence of 1 µM acetylcholine (ACh)-mediated dilation as described previously (Fig. 4a)^25–28^. Both endothelium-intact and -denuded vessels were preconstricted with 1 µM phenylephrine (PE). Application of 1 µM ACh fully reversed PE constriction in endothelium-intact arteries. In contrast, endothelium-denuded vessel showed only 5% reversal, which is 95% less than that shown by endothelium-denuded arteries (Fig. 4a,b). However, application of 1 µM sodium nitroprusside (SNP), a nitric oxide (NO) donor, fully reversed PE constriction in both endothelium-intact and -denuded vessels, suggesting that endothelium denudation does not affect smooth muscle responses to NO (Fig. 4b). 60K-induced vasoconstriction was also similar between endothelium intact and denuded arteries (Fig. 4c). Since ACh-induced vasodilation is primarily dependent on endothelial NO production, selective loss of ACh-evoked vasodilation demonstrates successful endothelium denudation. After validating endothelium denudation, we recorded neflamapimod-evoked vasorelaxation in both endothelium-intact and -denuded mesenteric arteries. Our myography data on endothelium-intact mesenteric arteries reveal that application of 10 µM neflamapimod dilated mesenteric arteries by ~ 43 µm (Fig. 4d,e). On the other hand, neflamapimod application on endothelium-denuded mesenteric arteries produced a dilation of ~ 38 µm, which is ~ 89% or similar, compared to endothelium-intact mesenteric arteries (Fig. 4d,e). This data suggests that endothelium denudation does not affect neflamapimod-evoked vasodilation.

**Figure 5:**
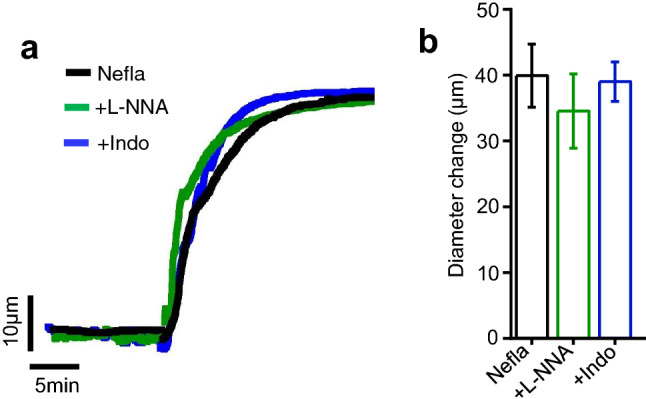
Role of endothelial NO and PGI_2_ in neflamapimod-induced vasodilation. (**a**) Original traces illustrating vasodilatory responses produced by neflamapimod (nefla, 10 µM) alone, and together with L-NNA (10 µM) or indomethacin (indo, 1 mM) in endothelium-intact mesenteric arteries. (**b**) Bar graph showing that the application of L-NNA and indo did not reduce neflamapimod-induced vasorelaxation in mesenteric arteries. n = 5.

Consistently, pharmacological inhibition of endothelial nitric oxide synthase (eNOS) with L-NNA and the production of the most important physiological vasodilator molecule NO do not reduce neflamapimod-evoked vasodilation (Fig. 5a,b), precluding the role of NO in this process. Likewise, neflamapimod-evoked vasodilation remained unchanged by the application of indomethacin which inhibits cyclooxygenase (COX) and synthesis of PGI_2_, another important endothelium-derived vasodilator (Fig. 5a,b). These data suggest that endothelial signaling has no role in acute neflamapimod-evoked vasodilation.

### Neflamapimod-induced vasodilation is not mediated by smooth muscle cell K^+^ channels

**Figure 6:**
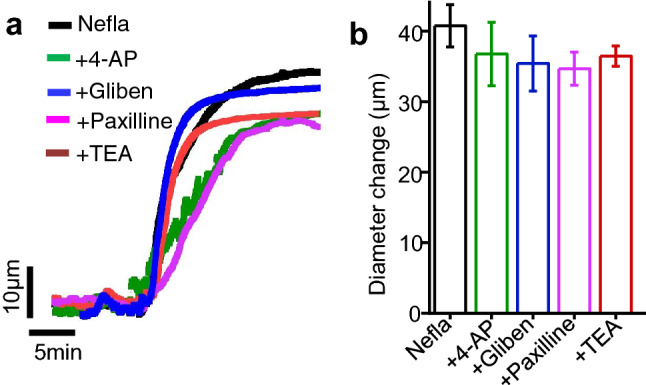
Role of smooth muscle cell K^+^ channels in neflamapimod-induced vasodilation. (**a**) Original traces comparing vasodilation induced by neflamapimod (nefla, 10 µM) alone, and together with 4-AP (1 mM) or glibenclamide (gliben, 10 µM), or paxilline (10 µM), or TEA (tetraethylammonium, 1 mM) in endothelium-intact mesenteric arteries. (**b**) Mean data showing that 4-AP, gliben, paxilline or TEA did not reduce neflamapimod-induced vasorelaxation in mesenteric arteries. n = 4.

Opening of arterial smooth muscle cell K^+^ channels and K^+^ efflux induce smooth muscle cell hyperpolarization, leading to vasodilation. Arterial smooth muscle cells express a variety of K^+^ channels including voltage-gated K^+^ channels (K_V_), large-conductance Ca^2+^-activated K^+^ channels (BK_Ca_), and ATP-sensitive K^+^ channels (K_ATP_). We therefore assessed the role of the major K^+^ channels in neflamapimod-induced mesenteric artery vasodilation. Application of 4-aminopyridine (4AP), an inhibitor of K_V_ channels, produced a mean dilation of ~ 37 µm, which is 90% of that produced by neflamapimod (Fig. 6a,b). Similarly, application paxillin, a BK_Ca_ channel blocker, and glibenclamide, a blocker of K_ATP_ channels, both relaxed mesenteric arteries by ~ 35 µm (Fig. 6a,b). Application of TEA, a general K^+^ channel blocker, dilated mesenteric arteries by ~ 36 µm, which is ~ 88% of that produced by neflamapimod (Fig. 6a,b). Overall, these data suggest that smooth muscle cell K^+^ channels do not mediate neflamapimod-induced vasodilation in resistance mesenteric arteries.

## Discussion

In this study, we demonstrate for the first time that acute neflamapimod application stimulates vasodilation in resistance-size mesenteric arteries. Our data suggests that neflamapimod reduces basal phosphorylation of p38 MAPKα and its downstream target Hsp27 to induce vasorelaxation. Our data also indicates that neflamapimod-evoked vasodilation is not dependent on endothelial signaling or smooth muscle cell K^+^ channels.

While the vast majority of previous studies focused on the role of p38 MAPKα in inflammation, its role in regulating arterial contractility remained virtually unexplored. Clues about the involvement of p38 MAPK(α/β) activity in arterial contractility surfaced from earlier reports of angiotensin II- and norepinephrine-induced phosphorylation of p38 MAPK and Hsp27, leading to vasoconstriction^7,23,24^. Hsp27 is an actin-associated protein that regulates actin cytoskeleton reorganization, which is a critical step in smooth muscle contraction. Vasoconstrictors such as angiotensin II, norepinephrine and endothelin-1 enhance Hsp27 phosphorylation and actin filament formation via p38 MAPK activation. Inhibition of p38 MAPK or disruption of cytoskeleton blocks or reverses these vasoconstrictor responses^29–31^.

Our finding is in agreement with previous studies in that selective p38 MAPKα inhibition, like non-selective p38 MAPK inhibition, reduces the phosphorylation of Hsp27. Reduced phosphorylation of Hsp27 decreases its actin capping ability, leading to smooth muscle relaxation. Komers et al. demonstrated that non-selective inhibition of α and β isoforms of p38 MAPK by SB203580 relaxed renal arteries and enhanced ACh-induced vasodilation^4^. However, whether selective inhibition of the α isoform of p38 MAPK could relax resistance arteries of the systemic circulation remained unknown. Our study provides pharmacological and biochemical evidence that selective inhibition of p38 MAPKα and downstream Hsp27 phosphorylation underlie neflamapimod-induced vasodilation in mesenteric arteries.

Neflamapimod at 10 µM concentration induced ~ 17% relaxation of mesenteric artery diameter from baseline. Therefore, such vasodilation upon acute neflamapimod application may lead to a significant drop in arteriolar resistance and systemic blood pressure in vivo. Mesenteric arteries are part of the splanchnic circulation that receive approximately 25% of cardiac output. The splanchnic circulation regulates the distribution of cardiac output and acts as a blood reservoir. Thus, vasodilation in small mesenteric arteries is likely to lower peripheral vascular resistance and systemic blood pressure. However, future studies will be required to investigate these possibilities.

Earlier studies suggest that non-selective inhibition of p38 MAPK improves endothelial dysfunction, enhances NO bioavailability and improves ACh-induced vasodilation, presumably by reducing vascular inflammation. Based on the findings in our study and previous reports, it is possible that neflamapimod treatment may improve long-term blood pressure control by reducing both vascular resistance and vascular inflammation.

Cherian et al.^32^, investigated the in vivo efficacy of a related p38 MAPKα inhibitor, losmapimod in hypercholesterolemic patients and found that a 7.5 mg twice daily treatment with losmapimod for 28 days improved endothelium-dependent vasodilation and forearm blood flow^32^. However, losmapimod treatment did not alter baseline vascular responses, which contrasts with the findings of our study. As hypercholesterolemia triggers a complex series of changes in the signaling landscape due to oxidative stress, it is likely that the aberrant signaling promotes both endothelial and smooth muscle dysfunction^32–35^. Upon exposure to oxidative stress and inflammation, smooth muscle cells undergo phenotypic switch from a contractile to a non-contractile, synthetic phenotype. While endothelial dysfunction could be reversed by treatment with a p38 MAPK inhibitor, this approach may not reverse altered smooth muscle contractility, especially after a short-term treatment period of 4 weeks. Since vascular contractility is regulated differently between different species, vessel types as well as in health and disease, the vascular effects of losmapimod on basal vascular reactivity remains unclear and will require future investigation.

Due to the involvement of p38 MAPKα in inflammation, there is great enthusiasm for developing selective p38 MAPKα inhibitors for the treatment of inflammatory diseases. As a selective inhibitor of p38 MAPKα, neflamapimod is currently being investigated for its efficacy in reversing neuroinflammation, neurodegeneration and cognitive deficits in DLB and AD. In clinical trials, neflamapimod treatment appears to be promising in reversing cognitive deficits in DLB and likely AD. Hypertension, through a variety of mechanisms, may lead to cognitive dysfunction in these patients. Proposed mechanisms by which hypertension leads to dementia include microvascular damage and dysfunction that results in white matter disease, microinfarcts, microhemorrhages, poor clearance of amyloid-β peptide among others^14,16,17^. In addition, elevated systemic blood pressure negatively impacts cerebral perfusion, which alters brain structure and function by promoting ischemic injury, oxidative stress and endothelial dysfunction^14^. Although hypertension and increased vascular tone are known drivers of cognitive dysfunction^14–17^, it remains unknown if neflamapimod treatment-associated improvement in cognitive function in DLB and AD could be due to a reduction in peripheral vascular resistance as a result of direct vasorelaxation of the systemic resistance vessels such as the resistance-size mesenteric arteries. Our finding of a direct vasodilatory effect of neflamapimod suggests that the observed clinical benefit with neflamapimod pharmacotherapy may result from direct vasodilation and reduction of vascular resistance, as well as the reduction of inflammation of the cardiovascular and nervous systems as proposed by others earlier.

This study laid the foundation for future studies that acute inhibition of p38 MAPKα could induce vasodilation, which in turn, could enhance regional organ blood flow. Our future study will examine if neflamapimod directly relaxes resistance cerebral arteries that control brain perfusion and cognitive function.

In conclusion, our study demonstrates that acute neflamapimod application relaxes resistance mesenteric arteries via selective inhibition of smooth muscle cell p38 MAPKα and the phosphorylation of its downstream target Hsp27. Inhibition of Hsp27 phosphorylation decreases its actin capping ability, resulting in smooth muscle relaxation and vasodilation. Neflamapimod-evoked vasodilation appears to independent of endothelial signaling and smooth muscle cell K^+^ channels. As elevated blood pressure and altered vascular tone contribute to cognitive dysfunction, vasodilatory effect of this drug is likely to have positive impact on the cognitive performance. We propose that the vasodilatory action of neflamapimod may be clinically translatable and is likely to improve systemic blood pressure and cognitive deficits in AD and DLB patients for which neflamapimod is currently being investigated.

## Materials and methods

### Drugs and chemicals

Neflamapimod (Nefla) was purchased from MedChemExpress (Monmouth Junction, NJ, USA) and used in the concentration range of 0.001–10 µM. SB203580 was purchased from MedChemExpress and used at a final concentration of 10 µM. Phenylephrine (PE), and 4-aminopyridine (4-AP) purchased from Sigma-Aldrich (St. Louis, MO, USA) were used at a final concentration of 1 µM and 1 mM, respectively. Glibenclamide (10 µM), paxilline (10 µM), nitro-l-arginine (L-NNA, 10 µM), sodium nitroprusside (SNP, 10 µM), indomethacin (indo, 10 µM) and acetylcholine (ACh, 1 µM)) were purchased from Tocris (Minneapolis, MN, USA). 4-AP, SNP, indo and PE were dissolved in distilled water. Nefla, gliben, paxilline, SB203580, L-NNA, and ACh were dissolved in dimethyl sulfoxide (DMSO). The maximum concentration of DMSO in the myograph chamber was < 0.1%, which had no effect on arterial contractility.

### Animals

Animal protocols were reviewed and approved (Ref. No. A2107011) by the Mercer University Institutional Animal Care and Use Committee (IACUC), and animal experiments were performed according to the guidelines set by the United States National Institutes of Health (NIH). We used male Sprague Dawley (SD) rats between 7 and 10 weeks of age. Rats were purchased from Charles River Laboratories (Wilmington, MA, USA) and acclimatized for 1 week at the Mercer University College of Pharmacy Vivarium prior to experimentation. Rats were individually caged in a temperature-regulated room (temperature 22 ± 2 °C; 55% humidity; 12-h light/dark cycles) and had access to standard chow diet and drinking water ad libitum. Animals were randomized for all experments^36^.

### Tissue preparation

CO_2_ inhalation was used as per the ‘2020 AVMA Guidelines for the Euthanasia of Animals’ to euthanize rats followed by decapitation. Entire mesenteric artery bed was dissected out and placed into ice-cold PSS containing (in mM) KCl 6.0, NaCl 112, NaHCO_3_ 1.18, MgSO_4_ 1.18, KH_2_PO_4_ 1.18, CaCl_2_ 1.8, and glucose 10. Third to fifth order branches of mesenteric arteries (150–250 µm) were dissected cleaned of adventitial tissue in ice-cold PSS. Cleaned arterial branches were cut into 1–2 mm long segments that were kept in ice-cold PSS until cannulated for pressure myography^25,26,28^. PSS for vessel dissection, isolation and preparation contained (in mM) KCl 6.0, NaCl 112, NaHCO_3_ 1.18, MgSO_4_ 1.18, KH_2_PO_4_ 1.18, CaCl_2_ 1.8, and glucose 10. pH of the PSS was adjusted to and maintained at 7.4 by gassing with a mixture of 21% O_2_, 5% CO_2_, and 74% N_2_. 60 mM K^+^-PSS (60 K) was prepared by equimolar replacement of NaCl with KCl in the PSS^36^.

### Pressure myography

Arterial segments were cannulated in a temperature-controlled perfusion chamber (Living Systems Instrumentation, St. Albans, VT), continuously perfused with 37 °C PSS, and gassed with mixture of 21% O_2_, 5% CO_2_, and 74% N_2_ to maintain pH 7.4. 60 mM K^+^-PSS (60 K) was prepared by equimolar replacement of NaCl with KCl in the PSS and used for checking the viability of the cannulated arteries at 10 mmHg. Using a pressure servo controller with peristaltic pump (PS-200, Living Systems) intraluminal pressure was gradually increased to 80 mmHg with increments of 20 mmHg at each step. Mounted arteries were maintained for at least 10 min at each pressure step so that the arteries reach a stable diameter. Mesenteric arterial segments developed a stable myogenic tone at 80 mmHg, after which a cumulative concentration response to neflamapimod (0.0001–10 µM) was performed^36^. Vessel diameter was read at 1 Hz using a charge-coupled device (CCD) camera connected to a Nikon Ts2 microscope and the automatic edge-detection function of IonWizard software (IonOptix, Milton, MA, USA). Myogenic tone (%) was calculated as: 100 × (1 – D_active_/D_passive_), where D_active_ is active arterial diameter in the presence of Ca^2+^ and D_passive_ is the arterial diameter determined in Ca^2+^-free PSS supplemented with 5 mM EGTA^25–28^. Endothelium denudation was performed by slowly passing air bubbles through the vessel lumen for 1–2 min. Arteries exhibiting at least 80% reduction of ACh-induced vasodilation were considered endothelium denuded^25–27^. Endothelium denudation selectively abolishes ACh-induced vasodilation without altering artery responses to sodium nitroprusside (SNP) and 60K^36^.

### Western blotting

Clean mesenteric artery branches were cut into small pieces and homogenized in modified RIPA buffer (50 mM Tris–HCl, 150 mM NaCl, 5 mM EDTA, 1% Nonidet P-40, 0.5% sodium deoxycholate, 0.1% SDS, 10 mM NaF, 10 mM Na_2_HPO_4_, pH 7.4) containing protease and phosphatase inhibitor cocktails (Roche). Arterial lysates were centrifuged at 12,000 rpm for 10 min at 4 °C and supernatants collected in clean eppendorf tubes. After measuring protein concentrations using BCA method, 40 µg protein for each sample was boiled with 2× SDS-sample buffer (Bio-Rad) for 5 min and resolved by SDS-PAGE. Proteins were transferred onto PVDF membranes using BioRad semidry transfer apparatus^26,37^. PVDF membranes containing transferred proteins were blocked with 5% milk solution in TBST (tris-buffered saline with 0.1% Tween 20) and incubated with the following primary antibodies overnight: phospho-p38 MAPKα (Thr180/Tyr182, 1:500 dilution, Thermo Scientific, Waltham, MA, USA), p38 MAPK (1:500 dilution, Cell Signaling Technology, Danvers, MA, USA), Hsp27 (1:200 dilution, Cell Signaling Technology), and phospho-Hsp27 (Ser82, 1:200 dilution, Cell Signaling Technology). Due to molecular weight overlap, phospho-p38 MAPKα/Hsp27 and total p38 MAPK/Hsp27 were probed in parallel using the same samples. For loading control, we used GAPDH antibody (Santa Cruz Biotechnology, Dallax, TX, USA at 1:1000 dilution). Following primary antibody incubation, blots were washed three times, 5 min per wash to remove unbound primary antibodies, and then incubated with HRP-conjugated secondary antibodies (Santa Cruz Biotechnology, 1:5000 dilution) for 1 h at room temperature. PVDF membranes were washed three times and developed using ECL (Pierce). Protein bands were detected using Gel Doc XR + System (Bio-Rad) and quantified by densitometry using ImageJ software (64-bit Java 1.8.0_112, National Institutes of Health, Bethesda, MD; URL: https://imagej.nih.gov/ij/download.html^26,38^. For reprobing, PVDF membranes were incubated in stripping buffer, washed, blocked in 5% milk solution, and processed for primary and secondary incubation as described before.

### Statistical analysis

OriginLab software version 9.55 (2018b, URL: https://www.originlab.com/index.aspx?go=SUPPORT&pid=3325) was used for statistical analysis. Values were expressed as mean ± standard error of mean (SEM). One-way analysis of variance (ANOVA) along with Newman-Keuls post-hoc test was used for multiple group comparison. p < 0.05 was considered statistically significant^26,38^.

## Supplementary Information

Supplementary Figures.
