# Supplementary material for: Neflamapimod induces vasodilation in resistance mesenteric arteries by inhibiting p38 MAPKα and downstream Hsp27 phosphorylation
Source: Sci Rep. 2022 Mar 22;12:4905. doi: 10.1038/s41598-022-08877-8 (PMC8941071; doi:10.1038/s41598-022-08877-8)
Supplement: Supplementary file 1 — Supplementary Figures. [file 41598_2022_8877_MOESM1_ESM.pdf]

Supplementary material

Neflamapimod induces vasodilation in resistance mesenteric arteries by inhibiting p38 MAPKα and downstream Hsp27 phosphorylation

Ajay K. Pandey[a#], Farzana Zerin[a#], Sreelakshmi N. Menon[a], Tanzia I. Tithi[a], Khue P. Nguyen[a], Tran Vo[a], Morgan M. Daniel[a], Sherif Hafez[a], Md. Ashraful Alam[b] , and Raquibul Hasan[a*]

[a]Department of Pharmaceutical Sciences, College of Pharmacy, Mercer University, Atlanta, GA 30341, United States

[b]Department of Pharmaceutical Sciences, North South University, Dhaka 1229, Bangladesh

***Correspondence:**
Raquibul Hasan, PhD, Department of Pharmaceutical Sciences, College of Pharmacy, Mercer University, 3001 Mercer University Drive, Atlanta, GA 30341, United States. Telephone: (678) 547-6223, Fax: (678) 547-6423, E-mail: hasan_r@mercer.edu

#equal contribution

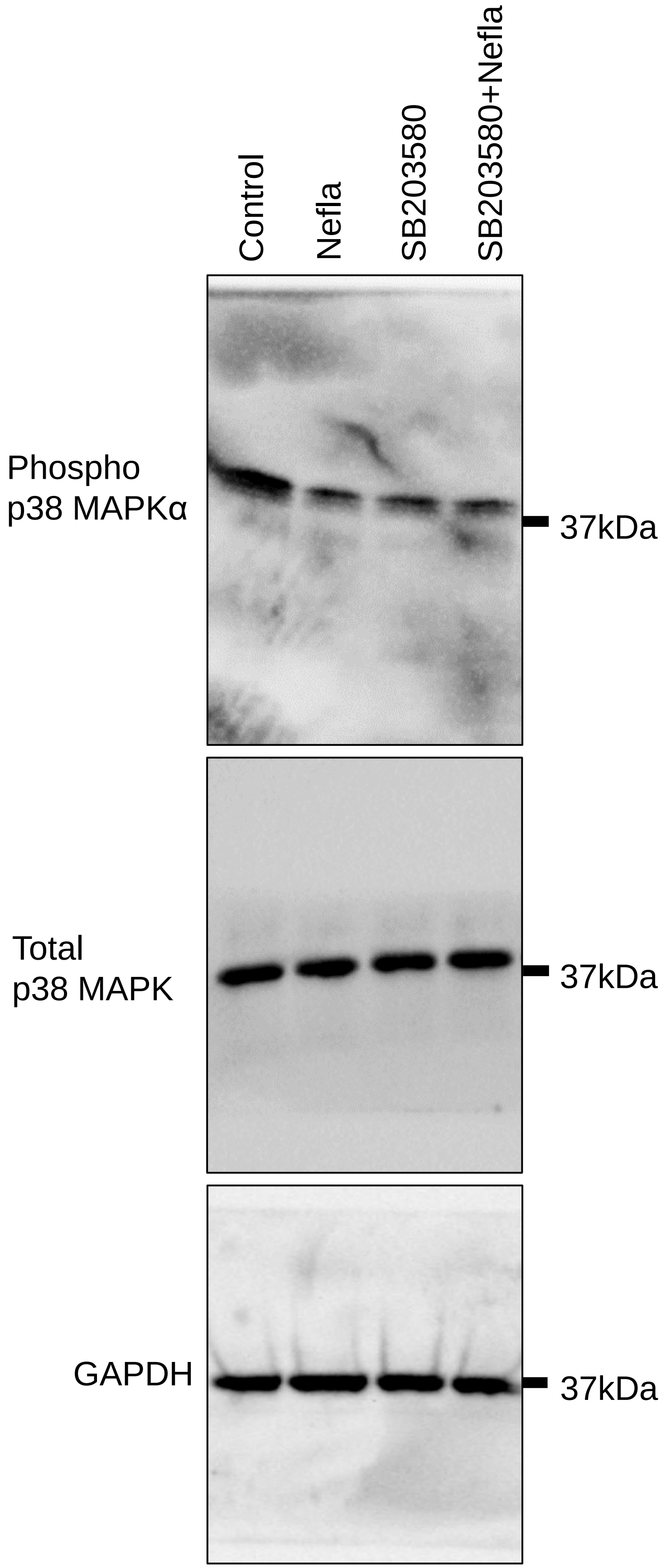

Supplementary Figure 1: Representative full-length Western blot images of phospho-p38 MAPKα, total p38 MAPK and GAPDH representing Western blot images in Figure 2.

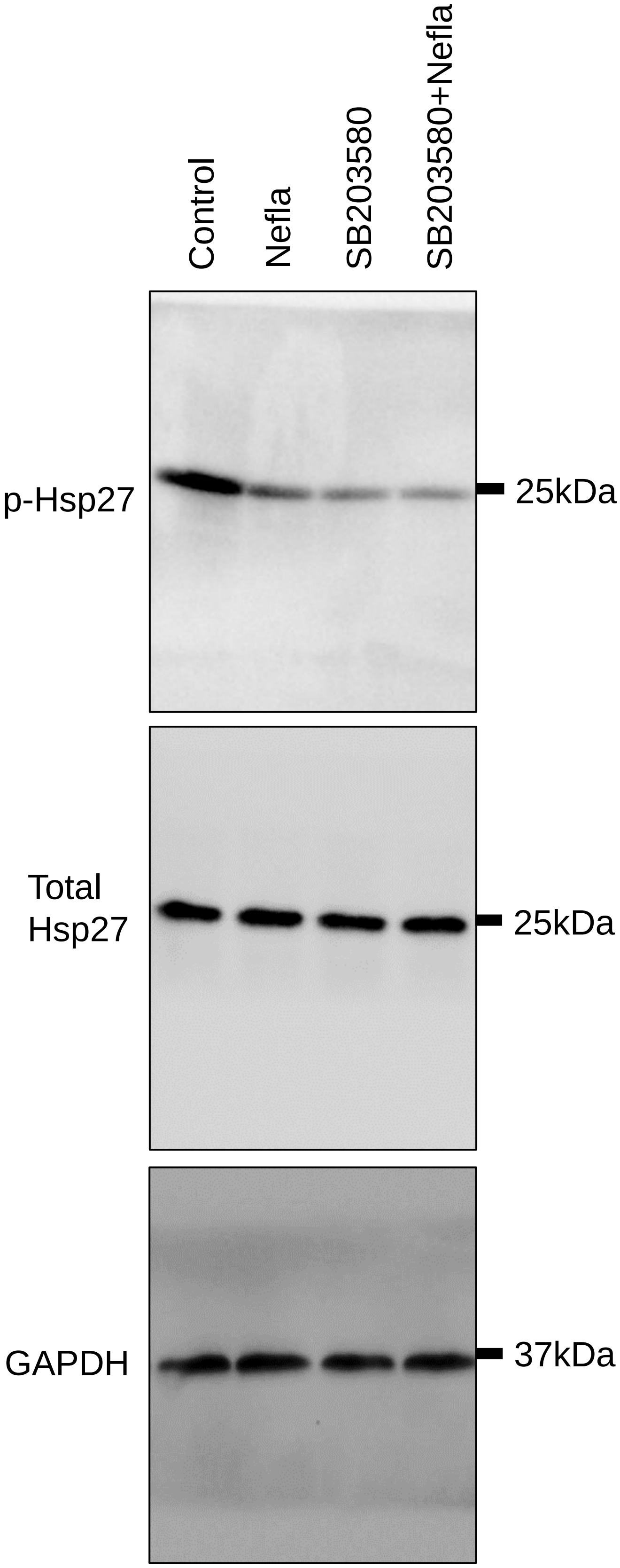

Supplementary Figure 2: Representative full-length Western blot images of p-Hsp27, total Hsp27 and GAPDH representing Western blot images in Figure 3.
